# The impact of financial stress on student wellbeing in Lebanese higher education

**DOI:** 10.1186/s12889-024-19312-0

**Published:** 2024-07-06

**Authors:** Ramona Nasr, Abir Abdel Rahman, Chadia Haddad, Nada Nasr, Joanne Karam, Jessy Hayek, Ibrahim Ismael, Eman Swaidan, Pascale Salameh, Nael Alami

**Affiliations:** 1 School of Health Sciences, Modern University for Business and Science, Damour, Lebanon; 2 Center of Excellence in Research, Education, and Cultural Studies (CEREC), Beirut, Lebanon; 3 Department of Liberal Education, Lebanese American University, Beirut, Lebanon; 4 Medical Laboratory Sciences Program, Faculty of Health Sciences, University of Balamand, Balamand, Lebanon; 5 Institut National de Santé Publique, d’Épidémiologie Clinique et de Toxicologie-Liban (INSPECT-LB), Beirut, Lebanon; 6 School of Medicine, Lebanese American University, Byblos, Lebanon; 7 Research Department, Psychiatric Hospital of the Cross, Jal Eddib, Lebanon; 8 School of Education and Social Work, Modern University for Business and Science, Damour, Lebanon; 9 Department of Natural Sciences, Lebanese American University, Beirut, Lebanon; 10 Faculty of Nursing & Health Sciences, Notre Dame University Louaize, American University of Beirut (AUB), Zouk Mosbeh, Lebanon; 11 Research Department, Modern University for Business and Science, Damour, Lebanon; 12 Department of Primary Care and Population Health, University of Nicosia Medical School, Nicosia, 2417 Cyprus; 13 School of Pharmacy, Lebanese University, Hadat, Lebanon

**Keywords:** Financial stress, University students well-being, Sleep quality, Psychological distress perceived stress, Lebanon

## Abstract

**Background:**

The financial crisis has indirectly affected Lebanese university students, leading to economic distress. Accordingly, this study aimed to assess the substantial negative impact of financial stress on the mental health and well-being of Lebanese college students.

**Methods:**

A quantitative research approach was applied and took place from June 13th to July 25th, 2023, targeting 1272 university students aged 17 and above from private and public universities across Lebanon through convenience sampling. The InCharge Financial Distress/Financial Well-Being scale (IFDFW), Pittsburgh Sleep Quality Index (PSQI), Beirut Distress Scale (BDS-10), Perceived Stress Scale (PSS-10), and Well-Being Index (WHO-5) were used to assess the students’ well-being. Descriptive analyses of the data was performed using SPSS software version 25.

**Results:**

1272 university students participated in this study, mostly females, with a mean age of 21.64 (± 4.43) years. Participants reported a lack of financial independence, unemployment, and no income. Positive associations were obtained between the BDS total scale as well as the PSS total and PSQI scores, while there was a significant negative relationship between IFDFW and PSQI scores. Those with a higher GPA, majoring in science/health and medicine, living in rural areas, and graduate students were linked to lower PSQI and BDS-10 scores. Financial aid and financial independence were associated with lower PSQI and BDS-10 scores. PSS-10 scores were higher among students majoring in science/health and medicine. Higher scores on the IFDFW scale correlated with lower BDS-10 and PSS-10 scores. In contrast, females had higher BDS-10 and PSS-10 scores. Scoring higher on the PSS-10 and PSQI scales, living off campus, or majoring in science/health and medicine, were associated with higher on the WHO-5 scale.

**Conclusions:**

A significant impact of financial stress on college students in Lebanon was obtained, affecting their well-being and mental health aspects. Marital status, gender, academic major, region of living, and financial independence also influences students’ experiences. Tailored support and further research are needed to address these multifaceted challenges.

## Introduction

College students frequently endure financial stress due to their credit score, unexpected financial emergencies, and financial restrictions, which tremendously impact their academic performance as well as their mental and physical health negatively [1]. Some students who are under a lot of financial strain might not have as many relatives or friends to turn to in times of need, which could lead them to feel pressured to drop out of school [2], whereas others have gradually been compelled to tolerate a larger share of the expenses of college by taking out greater loans and making budget cuts to pursue their college aspirations, which makes them subject to financial stress [3]. Studies suggest that the relationship between students’ financial stress and their academic performance is significant. [4]. In addition, college students’ mental wellness is acknowledged as a significant public health concern on a global scale. For example, in Australia, a study reveals that older college students, post-graduates with more life experience, appear to show more resilience than distressed undergraduate students since they have better life skills to cope efficiently with stressful situations including financial burdens [5]. The findings of another study conducted in Malaysia showed that financial prosperity can be reliably estimated by financial knowledge, financial competence, and financial behavior [6].

Hence, considering the elevated occurrence of mental health issues among the young adult population including college students, there is an enduring connection between socioeconomic status and mental well-being throughout one’s life [7]. A further insight indicates that financial stress, along with all the complicated conditions that might accompany it, remains directly tied to anxiety shown in college students despite the presence of family support [8]. In the Philippines, for instance, college students reveal that students with no financial stress experience positive life satisfaction unlike those who live in less privileged circumstances and who suffer from poverty [9]. The increase in financial stress, and academic and family stress increase as well as reporting significant symptoms such as worry, anxiety, sleep deprivation, and depression among college students [10]. Moreover, college students frequently endure financial stress due to various factors, including tuition fees, which significantly impact their academic performance and mental health. This stress is exacerbated by their living arrangements. Students living with family members, who may have lower financial expectations, often experience less financial strain compared to those living independently in dormitories or rented accommodations, where financial responsibilities are higher [11].

More recent studies found that students who are struggling financially often find it challenging to navigate their contacts with more financially secure peers, which commonly leads to feelings of isolation and embarrassment [12]. Thus, with the escalating costs of higher education, it becomes imperative to equip college students and their families with the knowledge to navigate tuition payments and balance between academics and employment, thus preventing dropouts triggered by financial stress [13].

In Lebanon, students in private universities typically incur higher tuition fees than the public university, which charge minimal fees. In fact, amid the ongoing crises that the country is facing, which raised the average everyday costs to approximately 20$ in 2023 [14], Sarkis (2020) states that many educational institutions suffer as a result of the economic crisis, the extraordinary layoffs of employees and workers, the worsening of the national currency rate, and the increased number of students who will not be able to afford their tuition [15]. Further, this study adds that many students will not be able to attend premium universities and will switch to less expensive ones which will affect the social diversity on campus and the harmony of various socioeconomic groups [15]. Due to this crisis, Lebanese students in private universities started taking the bare minimum of courses while some were left with few alternatives and turned to financial help while others dropped out to get a job and save money, yet some noted that if the US dollar parity further rises, they will ultimately quit [16]. Shifting to the Lebanese university which is more affordable adds extra pressure on the university which is already struggling due to low budget and lack of the ability to take in a large number of students, which does not offer a functional solution [15]. Consequently, Lebanese students are denied the transforming impact of education due to high tuition costs and scarce financial support [17]. Also, casual interactions with Lebanese university students revealed that the most prevalent issue behind this financial stress has been the mental discomfort of not knowing how they will continue their studies and what decisions the institution will make next [18].

Finally, as the impact of financial stress on the mental health of Lebanese college students is still unfolding, it is crucial to assess students’ well-being in Lebanon based on currently accepted and agreed-upon indicators, including emotional and behavioral indicators in a country enduring financial instability affecting college students’ well-being. Our ultimate goal is to inform interventions and support systems specifically tailored for this vulnerable population. Additionally, we aim to contribute to a broader understanding of how economic crises impact the health of young adults.

## Methodology

### Materials & methods

#### Study design & sampling method

A quantitative research approach was applied in this study, targeting university students aged 17 years old and above from different regions in Lebanon and registered in the Lebanese University and different private universities in Lebanon. We employed a convenience sampling method by disseminating an online survey via Google Forms across various social media platforms. The data collection phase took place from June 13th to July 25th, 2023. All university students and fresh graduates aged 17 years old and above were eligible to participate and no exclusion criteria were applied. A total of 1272 responses were collected.

#### Population & sample size

This study specifically targets university students aged 17 years and above who are enrolled in various private and public universities in Lebanon and from different programs, including Health and Medicine, Business, Engineering, and Arts. According to the Lebanese Ministry of Education, university students constitute approximately 12.2% of the total young adult population in Lebanon [14], which translates to almost 669,747 students out of the general population (reaching 5,489,733 in 2022) [19]. The final sample constituted a total of 1272 university students of both genders. Adopting a confidence interval of (95%) using the G-Power software version 3.0.10 showed that the sample size of 1272 was considered enough.

### Ethical considerations

Approval from the ethical committee of the Modern University of Business and Science (MU-20230612-43, July 12th, 2023) was received for this study. University students filled out an anonymous online survey, after reading the explanation of the topic and the written consent form ensuring their confidentiality and anonymity were protected and explicitly agreeing to take part in the study. The collected data were merely used for scientific and research purposes. Participants were informed that they could opt out of the study at any time by contacting the research team via email. Data from participants who chose to opt-out were promptly removed from the dataset to ensure their exclusion from the analysis.

### Study tool

The online survey was available and distributed in English. In the context of the present study, the structure of the survey included sociodemographic data, academic performance, and psychological and quality of life scales.

The sociodemographic questions focused on assessing the university students’ age, gender, marital status, financial independence, monthly income, source of income, employment status, place of living, education level, and performance. Additionally, the university’s performance was assessed through the valuation of Grade Point Average (GPA), self-reported by participants.

### Financial stress measures

The financial stress of university students was assessed using the InCharge Financial Distress/Financial Well-Being scale (IFDFW). It is a self-assessment tool with 8 questions that gauge a person’s perception of their financial well-being or distress. It assesses a person’s financial situation on a scale that ranges from complete lack of financial distress or the highest level of financial well-being to extreme financial distress or the lowest level. Each question on the scale is assigned a number from 1 to 10. The sum of the number of points for each of the eight items was obtained and the total was then divided by 8 to calculate a score. The final score ranged from 1 to 10, where 1 = overwhelming financial distress/lowest financial well-being and 10 = no financial distress/highest financial well-being [20]. In this study, the Cronbach’s alpha value was 0.924 [20].

Additionally, questions about financial aid and financial autonomy were asked to further characterize the financial situation of participants.

#### Well-being and mental health measures

##### Insomnia

To assess and measure the quality of students’ sleep over a one-month time frame, The Pittsburgh Sleep Quality Index (PSQI) was used. The tool is a 19-item scale focusing on sleeping habits and experiences during the last months. Numbers were assigned to the verbal markers of the PSQI scale items through a 4-point scale (0 to 3). The duration of sleep, sleep disturbance, sleep latency, day dysfunction due to sleepiness, sleep efficiency, overall sleep quality, and meds need to sleep are calculated and combined into seven component scores, then summed up to produce a final Global PSQI Score, which can range between 0 and 21. A lower total score denotes better sleep, while a higher total score denotes worse sleep [21].

##### Psychological distress

The Beirut Distress Scale (BDS-10) is utilized for evaluating mental and psychological distress. It consists of 10 items that solely focus on stress, on a 4-point Likert scale, with 0 denoting “never” and 3 denoting “very much.” The sum of 10 questions was calculated to obtain a score ranging from 0 to 30. Distress levels were categorized as low distress (scores = 0), moderate distress (scores between 1 and 8), and high distress(scores ≥ 9). It is an abbreviated version of the BDS-22, demonstrating a high level of internal consistency, as indicated by a Cronbach’s alpha coefficient of 0.954 [22].

##### Perceived stress

The Mental Health Outcomes (Perceived Stress Scale (PSS-10) is a 10-item scale created to evaluate participants’ self-reported levels of stress by examining their thoughts and emotions from the previous months. Each question is scored by respondents on a scale of 0 to 4, with 0 representing “never” and 4 representing “very often”. Questions 4, 5, 7, and 8 were reversed. The final score was obtained through a sum of scores (from 0 to 40) with higher scores indicating higher perceived stress: scores ranging from 0 to 13 would be considered low stress; scores ranging from 14 to 26 would be considered moderate stress; and scores ranging from 27 to 40 would be considered high perceived stress [23, 24].

##### Psychological well-being

The psychological well-being of students was evaluated using the WHO-5 Well-Being Index. It is a brief survey with 5 simple and non-invasive questions. Each question is scored by respondents on a scale of 0 to 5, with 0 representing “at no time” and 5 representing “all of the time”. The values of the five responses are added up to get the raw score. The raw score ranges from 0 to 25 and then is multiplied by 4 to get a final score; with 100 denoting the best possible quality of life, while 0 indicates the worst possible quality of life [25].

### Data analyses

Data were analyzed using SPSS software version 25. Descriptive analyses were performed using absolute frequencies and percentages for categorical variables and mean and standard deviations (SD) for quantitative measures. The sample was normally distributed as verified by the visual inspection of the histogram, while the skewness and kurtosis were below |1.96 [26].

Bivariate analyses were then conducted using the Student’s independent t-test to compare continuous variables in two groups and the ANOVA test to compare three or more means. Pearson correlation was used for linear correlation between continuous variables.

Linear regression analyses were conducted, taking the well-being, emotional, and mental health scales as the dependent variables; hypotheses and assumptions were met (normality of residues, linearity of the association, homoscedasticity and absence of collinearity). All variables that showed a *p* < 0.2 in the bivariate analyses were considered important variables to be entered in the model to eliminate potentially confounding factors as much as possible. A *p-value* of less than 0.05 was considered significant.

## Results

Table 1 provides an overview of the sociodemographic characteristics of the participants (*N* = 1272). Participants in the present study have a mean age of 21.64 (± 4.43) years, with the majority being females (70.5%). Almost all the participants were either single, widowed, or divorced (94.4%). The majority reported a lack of financial independence (70.4%), with 47.3% having no income, and 69.4% solely depending on parents or guardians as the primary source of individual income. Almost half of the participants received financial aid (55.6%). Most of the participants were unemployed (62.3%) and living off-campus, with parents/guardians (76.2%). 59.7% of participants were also living in urban areas. Students were almost equally distributed among the second (29.6%), first (23.1%), third (20.9%), and graduate academic years (18.4%), with very few participants being in their fourth (5.0%) or fifth (2.9%) academic years. Majors were diverse, where the highest percentage of participants were prominently enrolled in “Health and Medicine” (32.3%), and “Business” (21.0%), compared to other majors. Moreover, the mean GPA of the study participants was 2.70 out of 4 ± 0.80. In fact, the predominance of students from the Health and Medicine field could be attributed to the higher number of students enrolled in these programs at the participating universities. The absence of 6th-year students can be also explained by the structure of medical programs in Lebanon, where the 6th year often involves clinical rotations and internships, making it difficult for students to participate in surveys.

**Table 1:** Sociodemographic characteristics of the participants (*N* =1272)

Variables	*N* (%)
Gender	
Male	375 (29.5%)
Female	897 (70.5%)
**Marital status**	
Single/widowed/divorced	1201 (94.4%)
Married	71 (5.6%)
**Financially independent**	
Yes	376 (29.6%)
No	896 (70.4%)
**Monthly income**	
No income	602 (47.3%)
< 500 $	494 (38.8%)
500–1000 $	108 (8.5%)
> 1000 $	68 (5.3%)
**Source of individual income**	
Parents/Guardians	768 (69.4%)
Parents/Guardians plus business	89 (8.0%)
Own business	80 (7.2%)
Part-time employee	81 (7.3%)
Full-time employee	88 (8.0%)
**Financial aid**	
Yes	707 (55.6%)
No	565 (44.4%)
**Employment status**	
Full-time employee	165 (13.0%)
Part-time employee	315 (24.8%)
Not employed	792 (62.3%)
**Place of living**	
On campus	72 (5.8%)
Off-campus, with parents/guardians	943 (76.2%)
Off-campus, not with parents/guardians	68 (5.5%)
Off-campus, with a partner	95 (7.7%)
Off-campus, alone	50 (4.0%)
Other	10 (0.8%)
**Region of living**	
Urban	759 (59.7%)
Rural	513 (40.3%)
**Academic year**	
First	294 (23.1%)
Second	377 (29.6%)
Third	266 (20.9%)
Fourth	64 (5.0%)
Fifth	37 (2.9%)
Graduate students	234 (18.4%)
**Major of the study**	
Business	263 (21.0%)
Law	17 (1.4%)
Agricultural and Food Sciences	36 (2.9%)
Arts and Sciences	168 (13.4%)
Education	86 (6.9%)
Engineering	131 (10.5%)
Health and Medicine	404 (32.3%)
Sciences	141 (11.3%)
Other	6 (0.5%)
	**Mean ± SD**
**Age**	21.64 ± 4.43
**GPA**	2.70 ± 0.80

Table 2 reports the description of the scales used in the study, showing average values for all scales.

**Table 2:** Description of the scales used in the study

	Median	Mean	SD	Minimum	Maximum
**Pittsburgh Sleep Quality Index (PSQI total score)**	7.00	7.55	3.66	0.00	21.00
**Beirut Distress Scale (BDS-10)**	16.00	16.17	7.70	0.00	40.00
**Perceived stress scale (PSS-10)**	21.00	21.73	5.93	3.00	40.00
**WHO-5 Well-Being Index**	48.00	48.92	23.08	0.00	100.00
**InCharge Financial Distress/Financial Well-Being Scale (IFDFW)**	5.25	5.26	1.25	1.00	10.00

Table 3 shows the bivariate analyses taking the psychological scales as the dependent variables. Females reported a higher mean on the PSQI (7.70 ± 3.66), BDS-10 (17.32 ± 7.41), and PSS-10 (22.34 ± 5.89) scales, while reporting a lower mean on the WHO-5 index compared to male participants (52.83 ± 24.37; *p = < 0.001*). Moreover, those who are single/widowed/divorced reported a higher mean on the PSQI (7.60 ± 3.64) and BDS-10 (16.32 ± 7.69) scales, while married participants had higher means on the WHO-5 index (57.46 ± 21.97) than other marital statuses (*p = 0.003*).

**Table 3:** Bivariate analysis taking the psychological scales as the dependent variables

	PSQI	BDS-10	PSS-10	WHO-5 Well-Being Index
**Gender**				
Male	7.19 ± 3.63	13.44 ± 7.68	20.25 ± 5.78	52.83 ± 24.37
Female	7.70 ± 3.66	17.32 ± 7.41	22.34 ± 5.89	47.15 ± 22.26
*p-value*	**0.024**	**< 0.001**	**< 0.001**	**< 0.001**
**Marital status**				
Single/widowed/divorced	7.60 ± 3.64	16.32 ± 7.69	21.77 ± 5.96	48.42 ± 23.05
Married	6.71 ± 3.85	13.76 ± 7.36	20.98 ± 5.36	57.46 ± 21.97
*p-value*	**0.047**	**0.006**	0.277	**0.003**
**Employment status**				
Unemployed	7.30 ± 3.59	15.89 ± 7.62	22.20 ± 6.26	48.20 ± 23.52
Employed	7.97 ± 3.72	16.64 ± 7.80	20.95 ± 5.26	50.11 ± 22.31
*p-value*	**0.002**	0.092	**< 0.001**	0.187
**Major of the study**				
Science/ health and medicine	7.16 ± 3.54	16.42 ± 7.46	22.53 ± 6.35	46.34 ± 22.63
Other	7.84 ± 3.72	15.99 ± 7.86	21.12 ± 5.52	50.77 ± 23.24
*p-value*	**0.001**	0.322	**< 0.001**	**0.002**
**Financially independent**				
Yes	8.08 ± 3.89	16.84 ± 8.13	20.54 ± 4.85	52.33 ± 21.64
No	7.33 ± 3.53	15.89 ± 7.49	22.22 ± 6.26	47.52 ± 23.51
*p-value*	**0.002**	0.052	**< 0.001**	**0.002**
**Financial aid**				
Yes	7.94 ± 3.80	16.40 ± 7.62	21.77 ± 5.65	48.61 ± 22.44
No	7.06 ± 3.41	15.89 ± 7.79	21.67 ± 6.27	49.38 ± 24.01
*p-value*	**< 0.001**	0.239	0.788	0.597
**Region of living**				
Urban	7.85 ± 3.75	16.74 ± 7.84	21.77 ± 5.77	48.38 ± 22.63
Rural	7.11 ± 3.47	15.34 ± 7.41	21.67 ± 6.16	49.69 ± 23.70
*p-value*	**< 0.001**	**0.002**	0.768	0.358
**Place of living**				
On campus	6.73 ± 3.46	14.11 ± 6.92	21.00 ± 5.62	56.48 ± 23.44
Off campus	7.58 ± 3.64	16.28 ± 7.73	21.77 ± 5.96	48.40 ± 23.03
Other	10.80 ± 5.02	18.40 ± 7.42	21.80 ± 5.32	51.60 ± 18.22
*p-value*	**0.003**	**0.044**	0.562	**0.021**
**Monthly income**				
No income	7.50 ± 3.66	15.93 ± 7.64	22.22 ± 6.25	47.84 ± 24.02
< 500 $	7.93 ± 3.73	16.31 ± 7.24	21.28 ± 5.30	47.99 ± 21.27
500–1000 $	6.73 ± 3.22	17.08 ± 8.54	21.96 ± 6.20	54.68 ± 23.80
> 1000 $	6.49 ± 3.37	15.89 ± 9.74	20.20 ± 6.57	65.08 ± 22.50
*p-value*	**0.001**	0.508	**0.009**	**< 0.001**
**Academic year**				
First till third	7.73 ± 3.70	16.19 ± 7.65	21.75 ± 5.80	48.45 ± 22.31
Fourth and five	7.62 ± 3.81	17.51 ± 7.76	22.24 ± 6.40	45.83 ± 27.19
Graduate students	6.82 ± 3.31	15.53 ± 7.80	21.41 ± 6.26	52.28 ± 24.46
*p-value*	**0.003**	0.096	0.482	0.064
	**Correlation coefficient**	**Correlation coefficient**	**Correlation coefficient**	**Correlation coefficient**
**Age**	-0.016	-0.024	-0.054	0.098
*p-value*	0.566	0.393	0.054	**0.001**
**GPA**	-0.154	-0.101	-0.040	0.048
*p-value*	**< 0.001**	**0.001**	0.202	0.127
**InCharge Financial Distress/Financial Well-Being Scale (IFDFW)**	-0.101	-0.119	-0.211	0.142
*p-value*	**< 0.001**	**< 0.001**	**< 0.001**	**< 0.001**

Employed participants had a higher mean on the PSQI scale (7.97 ± 3.72), compared to unemployed participants who had a higher mean on the PSS-10 scale (22.20 ± 6.26). Students studying majors other than science/health and medicine had a higher mean on the PSQI scale (7.84 ± 3.72) and the WHO-5 index (50.77 ± 23.24), while those majoring in science/health and medicine had a higher mean on the PSS-10 scale (22.53 ± 6.35). Financially independent participants also had a higher mean on the PSQI scale (8.08 ± 3.89) and WHO-5 index (52.33 ± 21.64), while those who were financially dependent reported a higher mean on the PSS-10 scale (22.22 ± 6.26).

Participants who were living in urban areas had a higher mean on the PSQI (7.85 ± 3.75) and BDS-10 (16.74 ± 7.84) scales. Those who were living in places other than on or off campus, had a higher mean on the PSQI (10.80 ± 5.02) and BDS-10 (18.40 ± 7.42) scales, while participants living on campus had the highest mean among places living on the WHO-5 index (56.48 ± 23.44).

Participants with no income reported a higher mean on the PSS-10 scale (22.22 ± 6.25), compared to those earning < 500$/month who scored a higher mean on the PSQI scale (7.93 ± 3.73), while the WHO-5 index responses were mostly influenced by those earning > 1000$/month (65.08 ± 22.50). In addition, the PSQI was the only scale affected by the academic year of participants (*p* < 0.05), with students in their first to third university years scoring the highest mean among others (7.73 ± 3.70).

Furthermore, the age of participants was positively correlated with the WHO-5 index (*r* = 0.098). Significantly, the participants’ GPA was negatively correlated with the reported PSQI (*r* = -0.154) and BDS-10 (*r* = -0.101) scales. The IFDFW scale was negatively associated with the PSQI (*r* = -0.101), BDS-10 (*r* = -0.119), and PSS-10 scales (*r* = -0.211) while being positively correlated with the WHO-5 index (*r* = 0.142).

Multivariable linear regression taking the psychological scale and the WHO-5 well-being scale as the dependent variable was conducted (Table 4). When considering the insomnia scale as the dependent variable (Model 1), the regression analysis showed a significant positive relationship between the BDS total scale (Beta = 0.258) as well as the PSS total (Beta = 0.082) and PSQI scores. On the other hand, there was a significant negative relationship between IFDFW scores and PSQI scores (Beta = -0.260). Moreover, those with a higher GPA, majoring in science/health and medicine, living in rural areas, or being in their graduate university year were likely to decrease the PSQI scale score by 0.451, 0.671, 0.553, and 0.591 points respectively. In contrast, students receiving financial aid or being financially independent were likely to increase the PSQI scale score by 0.475 and 0.619 points respectively.

**Table 4:** Multivariable linear regression

Model 1: taking the PSQI as the dependent variable					
	Unstandardized Beta	Standardized Beta	*p*-value	Confidence interval	
				Lower Bound	Upper Bound
BDS total scale	0.258	0.482	< 0.001	0.226	0.290
GPA	-0.451	-0.098	< 0.001	-0.685	-0.218
Major of the study (science/ health and medicine vs. other*)	-0.671	-0.089	0.001	-1.053	-0.288
PSS total	0.082	0.129	< 0.001	0.043	0.120
Region of living (rural vs. urban*)	-0.553	-0.073	0.004	-0.925	-0.181
InCharge Financial Distress/Financial Well-Being Scale (IFDFW)	-0.260	-0.088	0.001	-0.407	-0.113
Financial aid (Yes vs. No*)	0.475	0.062	0.016	0.089	0.861
Financially independent (Yes vs. No*)	0.619	0.075	0.005	0.183	1.055
Academic year (graduate vs. first till third year*)	-0.591	-0.059	0.025	-1.107	-0.075
Variables entered in the model: Gender, marital status, employment status, major of the study, financially independent, financial aid, region of living, place of living, monthly income, academic year, GPA, and InCharge Financial Distress/Financial Well-Being scale (IFDFW)					
**Model 2: taking the BDS-10 as the dependent variable**					
Gender (female vs. male*)	4.017	0.269	< 0.001	3.138	4.897
GPA	-0.623	-0.073	0.017	-1.136	-0.111
Academic year (graduate vs. first till third year*)	-1.924	-0.103	0.001	-3.099	-0.749
Marital status (married vs. single*)	-2.481	-0.080	0.011	-4.384	-0.578
Region of living (rural vs. urban*)	-1.093	-0.077	0.011	-1.935	-0.251
InCharge Financial Distress/Financial Well-Being Scale (IFDFW)	-0.390	-0.071	0.020	-0.717	-0.062
Financially independent (Yes vs. No*)	1.859	0.122	< 0.001	0.916	2.801
Variables entered in the model: Gender, marital status, employment status, financially independent, region of living, place of living, academic year, GPA, and InCharge Financial Distress/Financial Well-Being scale (IFDFW)					
**Model 3: taking the PSS-10 as the dependent variable**					
InCharge Financial Distress/Financial Well-Being Scale (IFDFW)	-0.929	-0.196	< 0.001	-1.180	-0.678
Gender (female vs. male*)	1.848	0.142	< 0.001	1.155	2.542
Financially independent (Yes vs. No*)	-1.102	-0.085	0.002	-1.813	-0.391
Major of the study (science/ health and medicine vs. other*)	0.778	0.065	0.020	0.121	1.435
Variables entered in the model: Gender, employment status, major of the study, financially independent, monthly income, age, and InCharge Financial Distress/Financial Well-Being scale (IFDFW)					
**Model 4: taking the WHO-5 as the dependent variable**					
PSS-10	-1.669	-0.425	< 0.001	-1.875	-1.462
PSQI	-1.701	-0.274	< 0.001	-2.027	-1.375
Age	0.284	0.053	0.034	0.022	0.547
Place of living (off campus vs. other*)	-4.932	-0.055	0.025	-9.253	-0.611
Major of the study (science/ health and medicine vs. other*)	-2.480	-0.053	0.034	-4.773	-0.187
Individual monthly income (high vs. no income*)	6.546	0.051	0.040	0.290	12.802
Variables entered in the model: Gender, marital status, major of the study, financially independent, place of living, monthly income, academic year, age, InCharge Financial Distress/Financial Well-Being scale (IFDFW), PSQI and PSS.					

*Reference group

When taking the BDS-10 scale as the dependent variable, the results showed that students with higher GPAs decreased the BDS-10 scale by 0.623 points, being in their graduate university year decreased the BDS-10 scale by 1.924 points, being married decreased the BDS-10 scale by 2.481 points, or living in rural areas decreased the BDS-10 scale by 1.093 points =. Similarly, having higher scores on the IFDFW scale decreased the BDS-10 scale by 0.390 points. In contrast, females were more likely to score higher on the BDS-10 scale by 4.017 points compared to males, and financially independent students were more likely to score higher on the BDS-10 scale by1.859 points compared to financially dependent students (Model 2).

Considering the PSS-10 as the dependent variable, the results showed that participants with higher scores on the IFDFW scale were likely to decrease the PSS-10 scale score by 0.929 points. Financially independent students were to decrease the PSS-10 scale by1.102 points, in contrast to females, who were likely to increase the PSS-10 scale by 1.848 points, and students majoring in science/health and medicine, who were likely to increase the PSS-10 scale by 0.778 points(Model 3).

Taking the WHO-5 scale as the dependent variable, the results showed that those scoring higher on the PSS-10 and PSQI scales, as well as those who are living off campus, or majoring in science/health and medicine, were likely to decrease the WHO-5 scale score by 1.669, 1.701, 4.932, and 2.480 points respectively. Conversely, participants with higher ages were likely to increase the WHO-5 scale score by 0.284 points, similar to those with high monthly income, who were likely to increase the WHO-5 scale score by 6.546 points(Model 4).

## Discussion

This study highlighted the complex interplay of demographic factors with financial stress and their impact on Lebanese college students’ well-being. We found that Lebanon’s ongoing economic crisis has profoundly affected its youth, including college students. This crisis impacted various aspects of young adults’ lives, such as mental health, sleep patterns, academic performance, and overall well-being. We examined the multifaceted nature of the current situation on students’ well-being and mental health, considering factors like gender, marital status, employment, and academic factors, and combining surveys and validated scales. The multivariable regression model highlights the interconnectedness of a variety of factors. Financial distress, gender, major of study, and marital status have varying effects on the psychological scales (BDS-10, PSS-10) and the WHO-5 Well-Being Index.

### Well-being, perceived stress, psychological distress and financial status

In this study, a worse financial status, as measured by the IFDFW, was independently and significantly associated with higher insomnia, perceived stress, and distress; however, its effect on mental well-being was masked by insomnia and stress. This demonstrates the complex nature of financial stress and its impacts on the well-being of students, which cannot be attributed to a single factor alone.

These findings are in line with previous studies. Greater financial difficulties were related to increased depression, anxiety, and mental health, with the economic recessions greatly impacting financial well-being and consequently mental health, which calls for proactive prevention [27–29]. Benson-Egglenton (2019) also found that students with lower well-being were more inclined to worse financial circumstances [27]. However, few studies have considered the association between financial factors and well-being directly. Park et al.’s (2017) study showed that financial hardship is correlated with perceived stress indirectly based on perception of situation, through intensifying the negative perceptions of one’s financial situation and elevating levels of perceived stress [30]. However, perceived stress was found to also mediate the relationship between financial strain, psychological symptoms, and, academic and social integration [31]. Psychological distress was also found to be affected by an individual’s perceived financial situation [32–34].

Lebanon has several public health interventions aimed at supporting mental health and well-being, including counseling services in universities, national mental health programs, and NGO-led initiatives providing psychological support. Despite these efforts, financial stress remains a significant issue among students [17]. Moreover, while the literature does not explicitly investigate the direct link between financial stress and sleep quality, our findings suggest that financial distress could potentially exacerbate sleep disturbances. This is the result intersection of financial stress and psychological distress can create a detrimental cycle, potentially leading to the poor sleep quality reported by many students. This indirect link further underscores the importance of exploring these complexities further. By doing so, we can advance our understanding of how financial stress affects students’ academic journeys and quality of life. This knowledge can inform the development of interventions and support systems tailored to the unique needs of these students, ultimately enhancing their well-being during their rigorous educational pursuits.

### Well-being, perceived stress, psychological distress, and financial independence

Our study has uncovered intriguing associations between financial independence and various dimensions of well-being among college students, including sleep quality and perceived stress levels. Notably, our findings indicate that financial independence is linked to poorer sleep quality, lower perceived stress levels, and higher overall well-being scores. These significant associations prompt us to consider potential explanations within the context of the college experience.

Financial independence, characterized by students managing their own finances without external support, may introduce unique stressors into their lives. The responsibility of tuition fees, living expenses, and other financial obligations may create pressures that impact sleep patterns [35]. It is possible that students who are financially independent face the burden of juggling work and academics to meet these financial demands, subsequently affecting their sleep quality.

On the contrary, our finding of lower perceived stress levels among financially independent students might be attributed to a sense of control over their financial situation. Financially independent students may have developed effective coping strategies and budgeting skills, reducing their overall stress levels. Additionally, these students may feel a greater sense of accomplishment and self-efficacy in managing their finances, leading to lower perceived stress.

While our study provides valuable insights, it is essential to acknowledge the limited availability of existing literature that directly addresses these connections between financial independence and sleep quality or perceived stress among college students. As such, these significant findings underscore the need for further research to unravel the intricate dynamics of financial independence within the college context. Future investigations can delve into the specific mechanisms and coping strategies that underlie these associations, shedding light on how to support financially independent students effectively.

### Well-being, perceived stress, psychological distress, and financial aid

In exploring the relationship between financial aid, and overall well-being in college students, we have come across an insightful finding. Our study revealed a significant association between financial aid status and sleep quality, with students receiving financial aid reporting poorer sleep quality compared to their counterparts. While this result is significant, it also raises questions about the underlying factors contributing to this connection.

While financial aid plays a vital role in supporting students’ educational pursuits, our study suggests that receiving financial aid may negatively impact students’ sleep quality. Several potential mechanisms may explain this relationship. One potential explanation is that acquiring financial aid is accompanied by added pressure and responsibility. Students who rely on financial aid often face the constant concern of maintaining certain academic performance standards to retain their aid. This can create a persistent source of stress due to the fear of losing their financial aid status potentially leading to sleep disturbances and poorer sleep quality.

It is crucial to acknowledge that the existing literature exploring the relationship between financial aid and sleep quality among college students is remarkably limited. Our study addresses a gap in this area by providing initial insights into this complex interplay and underscores the importance of further exploration in this domain. Educators should reconsider the potential negative academic effects and develop interventions to prevent severe financial hardship and reduce dependency on loans.

### Well-being, perceived stress, psychological distress and gender

The findings of this study regarding gender disparities and their impact on well-being are consistent with existing research on this subject. Remarkably, these studies consistently found that females tend to report higher levels of stress compared to their male counterparts [8, 36, 37], similar to our findings.

Moreover, research has consistently demonstrated that women are often at a higher risk of experiencing poorer sleep quality compared to men [35, 38, 39]. This aligns with our observation that male students in our study reported considerably better sleep quality. Interestingly, numerous studies have underscored the intricate relationship between stress and sleep quality, highlighting how heightened stress levels can lead to a decline in overall sleep quality, especially among women [35], graduate and undergraduate students [40, 41]. This phenomenon creates a potentially vicious cycle wherein female students, who are at a higher risk of both financial stress and poorer sleep quality, may find themselves facing compounding challenges in managing their overall well-being.

These disparities emphasize the need for gender-sensitive interventions and support mechanisms tailored to the unique challenges faced by female college students. As we navigate these challenging times, it is imperative for universities, policymakers, and public health practitioners to recognize the profound implications of financial stress on the well-being of college students, regardless of gender, and to take proactive measures to support and empower them on their academic journey. These measures could include providing financial literacy education and mental health resources to all students, regardless of gender, to mitigate the detrimental effects of financial stress.

### Well-being, perceived stress, psychological distress, and marital status

Our findings indicate notable differences in the well-being of college students based on their marital status, with married students reporting less distress and better well-being. Similarly, marital status is an essential aspect of an individual’s life that can influence various dimensions of well-being, including coping with distress, and overall satisfaction [42]. This suggests that marriage may provide emotional and financial support that contributes to a better overall sense of well-being among college students. Previous research has consistently shown that marriage can provide emotional stability and financial security, which are crucial factors in enhancing well-being among college students [39, 40]. For example, a study by Braithwate and colleagues (2010) revealed that college students in committed relationships experienced fewer mental health problems [43]. Interestingly, Uecker (2012) reported that married young adults were much more satisfied with their lives due to several explanatory factors which included relationship stability and psychological gains [44]. The author explained that the sense of satisfaction derived from marriage is due to the degree of certainty and accomplishment that married individuals reach marking the end of their transitionary period in adulthood. However, it is important to note that all studies emphasized that the quality of marriage had an impact on well-being and satisfaction. Interestingly, the study indicated no significant difference in sleep quality between single and married students. For that, it is essential to conduct further research to further explore these relationships.

### Well-being, perceived stress, psychological distress and major of study

Our findings closely align with the abundance of current research, which identifies many sources of stress among medical, science, and health students that consequently lead to significantly higher levels of psychological distress [45–48]. Medical students, in particular, often find themselves facing a distinct set of challenges, including financial ones. The substantial costs associated with medical education, such as tuition fees and student loans, can create a heavy financial burden. Consequently, medical students are put at an increased risk of psychological distress which has been reflected in the higher PSS-10 scores in our study population, linked to a lower well-being. This is due to the persistent worries that students might have regarding educational expenses, living costs, and future financial obligations can take a toll on mental and emotional well-being [42, 43]. Furthermore, our study aligns with abundant literature indicating that medical students frequently experience poorer sleep quality [49–51]. The high demands and stress associated with medical training may contribute to disrupted sleep patterns and, consequently, adverse impacts on overall health.

### Well-being, perceived stress, psychological distress and region of living

Our study sheds light on an intriguing dimension of stress concerning students’ geographical location. Urban students, in particular, tend to exhibit higher PSQI scores, suggesting poorer sleep quality, while their rural counterparts tend to have lower BDS-10 scores, indicating lower psychological distress. The existence of these disparities elicits us to explore the interlinkages between factors that may underlie this phenomenon. It is probable that urban students face a unique set of challenges, including the high cost of living, increased competition for resources, and a potentially more demanding lifestyle. According to Mato and Tsukasaki (2019) within urban settings, often characterized by abundant information and numerous resources, individuals are more likely to experience stress as a result of extensive networks [52]. A previous study also indicated that people residing in urban areas were more vulnerable to social stress compared to those in a non-urban area and that this inclination was stronger among individuals residing in larger urban areas and those who had spent a longer duration in urban environments during infancy [53]. These factors could contribute to heightened stress levels and poorer sleep quality among urban-dwelling students. However, in their study, Mojs et al. (2015), found no association between university students’ permanent place of residence and depression symptoms [54], which calls for further investigation.

### Well-being, perceived stress, psychological distress, and academic year

Our findings align with the current literature, revealing that students in their early academic years tend to experience poorer sleep quality, as evident in their higher Pittsburgh Sleep PSQI scores. This resonates with existing research indicating that the transition from high school to college marks a significant shift for undergraduates [55]. During this adaptation period, students are often confronted with a myriad of new stressors related to independent living, forging new relationships, heightened academic demands, and financial concerns [56].

On the other hand, an intriguing finding from our study is that graduate students report better sleep quality. This observation challenges the existing narrative as previous studies contradicted our findings, and it was uncommon to conclude that graduate students reported such a positive trend in sleep quality [57]. However, our findings offered an alternative perspective. These findings could be attributed to multiple factors experienced by graduate students, including better sleep hygiene, the ability to manage time and responsibilities, and relationship status [58].

### Practical implications

The financial stress experienced by Lebanese students is similar to that in other low- and middle-income countries (LMICs), such as Egypt and India, where economic instability and high educational costs also significantly impact students’ well-being [59, 60]. This comparison underscores the need for targeted financial aid and mental health support. Overall, the findings emphasize the need to alleviate financial stress among college students. It is critical that educational institutions and authorities give assistance and resources to help ease financial stress. In this context, financial aid programs, counseling services, and financial literacy courses can all be beneficial. Furthermore, the study emphasizes the importance of additional research into the details of the links between financial stress, gender, marital status, academic area, financial independence, and geographical characteristics. By acquiring a better knowledge of these linkages, more tailored interventions to assist college students’ well-being and academic achievement may be devised.

### Limitations

Several limitations should be acknowledged in this study. Firstly, a significant limitation of this study is the language barrier, as the questionnaire was distributed solely in English. This decision was made to ensure consistency and because English is commonly used in Lebanese higher education. However, it may have excluded non-English speakers and may have introduced a language barrier for some participants whose primary language is Arabic, Lebanese, or French, leading to a selection bias. Another limitation is the disproportionate representation of female respondents (70.5%), which does not accurately reflect the gender distribution of the Lebanese population. This could influence the generalizability of the results. Moreover, the questionnaire did not include an option for participants to indicate funding through a scholarship, which is a notable limitation as it might have overlooked an important aspect of financial support for some students. We note that questions related to financial difficulties could be considered delicate and subject to information bias, which might also affect the current results. Future studies should include this option to capture a more comprehensive view of students’ financial situations.

## Conclusion

The study found a significant impact of financial stress on the well-being and mental health of Lebanese college students. Specific factors such as marital status, gender, academic major, region of living, and financial independence was influential, emphasizing the need for tailored support. Accordingly, to enhance students’ well-being, targeted interventions and further research are essential in addressing these multifaceted challenges.

## Data Availability

The datasets generated during and/or analyzed during the current study are available from the corresponding author on reasonable request.

## Abbreviations

GPA: Antimicrobial Resistance
IFDFW: InCharge Financial Distress/Financial Well-Being scale
PSQI: Pittsburgh Sleep Quality Index
BDS: Beirut Distress Scale
PSS: Perceived Stress Scale
WHO: World Health organization
SPSS: Statistical Package for Social Sciences
SD: Standard Deviations
